# Recent progress on the molecular pharmacology of propofol

**DOI:** 10.12688/f1000research.12502.1

**Published:** 2018-01-29

**Authors:** Pei Tang, Roderic Eckenhoff

**Affiliations:** 1 Department of Anesthesiology, University of Pittsburgh, 3550 Terrace Street, Pittsburgh, PA, 15261, USA; 2 Department of Anesthesiology & Critical Care, University of Pennsylvania, 3400 Spruce St, Philadelphia, PA, 19104, USA

**Keywords:** propofol, ligand-gated ion channels, GABA, voltage-gated ion channels

## Abstract

The precise mechanism by which propofol enhances GABAergic transmission remains unclear, but much progress has been made regarding the underlying structural and dynamic mechanisms. Furthermore, it is now clear that propofol has additional molecular targets, many of which are functionally influenced at concentrations achieved clinically. Focusing primarily on molecular targets, this brief review attempts to summarize some of this recent progress while pointing out knowledge gaps and controversies. It is not intended to be comprehensive but rather to stimulate further thought, discussion, and study on the mechanisms by which propofol produces its pleiotropic effects.

## Introduction

Propofol is now the most commonly used general anesthesia induction agent in the world and is being used for total intravenous general anesthesia and sedation with increasing frequency. It is well tolerated and may be associated with fewer post-anesthesia effects than many of our other general anesthetics, factors that have undoubtedly contributed to its entrenched position in various clinical applications. However, propofol has adverse effects, including pain on injection, hypotension, hypoventilation, bradycardia, and hyperlipidemia ^1^. Aside from clinical usage, propofol is being used extensively in basic neuroscience research to better understand consciousness, memory, and learning ^2– 4^. For example, a recent study reveals a previously unknown mechanism of unconscious memory under propofol anesthesia, suggesting that general anesthesia acts at stages beyond cellular coding to disrupt sensory integration for higher-order association ^2^.

The success of this drug is all the more remarkable given how little we know of its full spectrum of molecular targets and actions. Although it has long been considered to be dominantly γ-aminobutyric acid-ergic (GABAergic) ^5, 6^, recent research has indicated that it is not exclusively so and in fact may rely on other molecular targets to produce even its principal desired effect: unconsciousness or hypnosis. This review will briefly cover recent research on how propofol functions on ligand-gated ion channels and then cover other recently revealed molecular targets.

## Ligand-gated ion channels

Pentameric ligand-gated ion channels (pLGICs), particularly GABA type A receptors (GABA _A_Rs), have been extensively investigated as molecular targets for propofol in the past ^7– 9^. Propofol typically inhibits cation-selective pLGICs, including the nicotinic acetylcholine receptor (nAChR) ^10– 13^ and prokaryotic homologues from *Gloeobacter violaceus* (GLIC) ^14, 15^ and *Erwinia chrysanthemi* (ELIC) ^16, 17^. In contrast, propofol at clinical concentrations potentiates agonist-evoked currents of the anion-selective GABA _A_Rs and glycine receptors (GlyRs) and increases the frequency of channel opening ^5, 6, 18, 19^. Propofol can also directly activate GABA _A_Rs at intermediate concentrations, but then inhibit conductance at high, supra-clinical concentrations ^6, 19^. Exploration of the structural basis of propofol action on pLGICs is still at an early stage. Only a few crystal structures or cryo-electron microscopy structures of eukaryotic pLGICs have been determined ^20– 24^, and none of these structures includes anesthetics. In contrast, propofol and other general anesthetics as well as alcohols have been successfully co-crystallized with the prokaryotic GLIC ^25, 26^ and ELIC ^17, 27– 29^.

The crystal structure of GLIC in a presumed open/desensitized state shows propofol localized to an intra-subunit pocket at the extracellular end of the transmembrane domain α-helices ^25^. Photoaffinity labeling of a propofol analogue to purified GLIC in solution leads to the same propofol location as observed in the crystal structure ^30^. This *intra-subunit* transmembrane pocket is probably a common anesthetic-binding site for *inhibition* of pLGICs. For example, desflurane binds close to this location in GLIC ^25^, and propofol inhibits ELIC via binding to the equivalent site ^31^. Similarly, propofol ^32^, etomidate ^33, 34^, and halothane ^35^ also bind to an intra-subunit site within the δ subunit helix bundle of the eukaryotic nAChR, another pLGIC that is inhibited by anesthetics.

A significant role of β3GABA _A_R in behavioral effects of propofol and etomidate has been demonstrated by the reduced sensitivity of β3(N265M) mice ^36^, and considerable progress has been made in revealing the underlying mechanisms ^36– 43^. Two recent articles ^42, 43^ have described sites for propofol in expressed αβGABA _A_R, albeit with somewhat different locations. In one case, *ortho*-propofol diazirine ( *o*-PD) was found to adduct to a β3 residue at the transmembrane/extracellular domain interface ^43^ while azi-propofol *meta* (aziP *m*) formed an adduct with residues deeper in the α1β3 transmembrane domain interface ^42^. Differences are likely due to different photochemistry of *o*-PD and aziP *m*. The less thermally stable *o*-PD is thought to undergo more complex chemistry on illumination and produce a longer-lived reactive intermediate than the carbene generated by illumination of aziP *m*. This more stable and likely more hydrophilic *o*-PD photoproduct then has more time to seek preferential photochemistry partners, such as the β3 histidine267 residue that may line the pore. A means of distinguishing such photochemical artifacts from specific binding is an ability of the parent drug to inhibit or “protect” the candidate site from photoadduction. Thus, aziP *m* photoadduction was indeed inhibited by propofol, but it is not reported whether *o*-PD was. Another important difference is the milieu in which the receptor resides during photolabeling. For the aziP *m* studies, the expressed αβ GABA _A_R resides in a detergent/lipid mixture, but the *o*-PD studies were performed with the recombinant receptor in *Spodoptera frugiperda* (Sf9) cell lipid membranes. Neither milieu is perfectly matched to the native neuronal membranes in which GABA _A_R normally resides. Thus, further studies will be required to give confidence that these sites are indeed physiologically relevant.

There are often multiple anesthetic-binding sites for a given anesthetic in a particular protein ^31, 34, 42, 44– 46^. The existence of multiple anesthetic sites has introduced a more challenging question: which specific site or sites among all those identified are responsible for the functional modulation? An effective way to answer this question is to use chimeras containing domains from different channels that have opposite responses to anesthetics. For example, photolabeling of ELIC with the light-activated derivative of propofol, aziP *m* ^47^, identified multiple aziP *m*-binding sites in the extracellular domain and one intra-subunit site in the transmembrane domain ^31^. To determine the functionally relevant propofol-binding site(s), we constructed an ELIC–GABA _A_R chimera ^16, 28, 31^ that contains the ELIC extracellular domain and the transmembrane domain of α1β3GABA _A_R. In contrast to inhibiting ELIC, propofol potentiates the ELIC–GABA _A_R chimera as it does on α1β3GABA _A_R. These results support a functionally dominant propofol-binding site in the transmembrane domain of ELIC ^16, 31^. In heteromeric GABA _A_Rs, propofol binds to multiple allosteric sites at the transmembrane β–α and α–β interfaces ^37, 42^. Both potentiation and direct activation of GABA _A_Rs are mediated by the same propofol-binding sites ^48^, but it is not clear whether one site plays a more critical role than others. It is also important to realize that propofol binding affects, not only channel activity but also receptor assembly and trafficking ^49, 50^.

A homomeric pLGIC, such as GLIC, has five identical subunits and five equivalent binding sites of each type; the crystal structure shows five propofol molecules bound symmetrically in the transmembrane domain ^25^. Is full occupancy of all five sites necessary for functional modulation? Molecular dynamic simulations suggest that asymmetric binding of propofol to only one, two, or three GLIC sites accelerated channel dehydration, increased conformational heterogeneity of the pore-lining helices, and shifted GLIC toward a closed-channel conformation ^51^ as compared with symmetrical (five-site) occupancy. Similarly, in homo-pentameric pLGICs, occupancy of from one to three sites by anesthetics has been found to be sufficient to potentiate channel currents ^52– 55^. Thus, it appears that maximum functional effects are produced by asymmetric occupancy of these channels.

The effect of propofol binding on channel function is largely determined by the intrinsic dynamics of the channel. The cation-conducting GLIC is inhibited by clinically relevant concentrations of general anesthetics ^14, 15^. The introduction of three mutations at the selectivity filter and one at the hydrophobic gate converted wild-type (wt) GLIC into GLIC4, an anion channel ^15^. None of the mutated residues is within the propofol-binding pocket, so propofol binding is probably the same in both GLIC4 and wtGLIC ^25, 30, 31^. Nevertheless, propofol is unable to inhibit GLIC4 as it does wtGLIC ^15^. Molecular dynamic simulations revealed that, compared with wtGLIC’s pore, GLIC4’s pore was more resistant to perturbation from propofol binding ^15^. These results underscore the importance of pore dynamics and conformation in ligand modulation of channel functions.

## Other molecular targets of propofol

**Figure 1.**
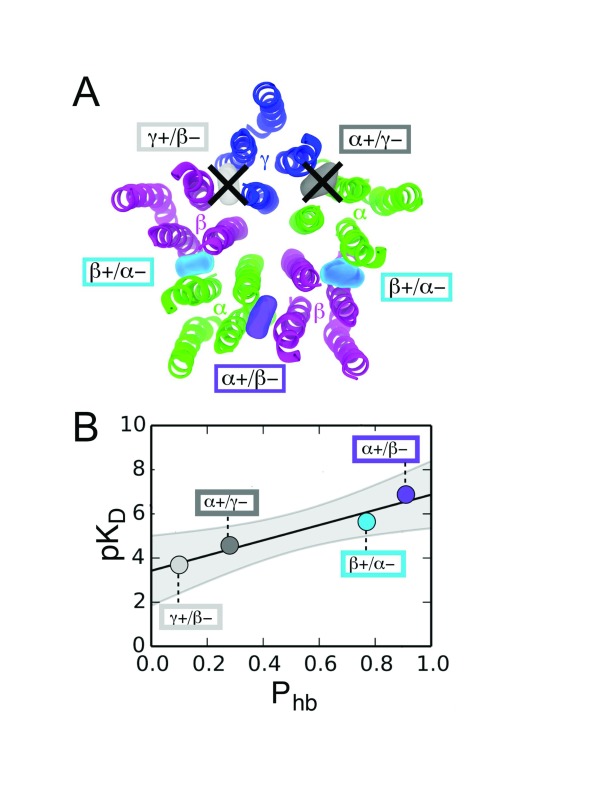
Asymmetric occupancy of the αβγGABA _A_ receptor by propofol. ( **A**) Photolabeling has identified interfacial binding sites for propofol (colored blobs) in the transmembrane region (seen from the extracellular view here). Furthermore, several lines of evidence now suggest that asymmetric occupancy of these sites confers a larger change in activity than symmetric occupancy (all five subunits). Click-enabled propofol analogues have confirmed asymmetric occupancy of αβγGABA _A_ receptor sites in their native, unperturbed state in that only α and β subunits were photoadducted. The mechanism in the case of this heteropentamer is differential affinity of the interfaces. ( **B**) The relationship between H-bond probability (P _hb_) and affinity (pK _D_) from molecular dynamic simulations shows where each interfacial binding site lies. The two γ-containing interfaces have a much lower P _hb_ and therefore lower affinity.

The search for other molecular targets has occurred in three principal ways. The first approach examined the transcriptomic, proteomic, or metabolomic response to propofol exposure in cells or intact animals. Depending on the analysis of the data, this approach has led to hundreds of potential molecular substrates ^56^. Because it is rarely clear whether, or how many of, these substrates are actually direct molecular binding targets of propofol, additional studies are required; some have been reported. In the second approach, propofol was altered to allow it to covalently bind to its targets on exposure to ultraviolet light (photolabeling), thereby serving as a tag. Tagged proteins then are discovered by using a variety of other technologies. In some cases, the tag is a radiolabel on the altered anesthetic, and scintillation counting and mass spectrometry (MS) are used to detect and identify adducted proteins. More commonly today, the tag is simply the additional mass of the adduct, and tagged proteins can be identified with shotgun MS approaches. The Achilles heel of this approach is that the identified targets are heavily biased toward the most abundant proteins, such as mitochondrial complexes, tubulin, and voltage-dependent anion channels ^57^. In fact, known targets of propofol, such as GABA _A_R (above), were not detected and this is presumably because of their low abundance. Thus, in the third approach, photolabeling approaches were refined to include another functional group on the aziP *m* molecule, in this case an alkyne (in place of the remaining isopropyl), in order to attach subsequent groups after photolabeling by using “click” chemistry ^58^. Remarkably, this heavily modified clickable photoactive derivative of propofol was still a potent general anesthetic and was still GABAergic ^59^. Deploying this “aziP *m*-click” compound involved first photolabeling in the presence of synaptosomes and then using click chemistry to attach a biotin moiety to the propofol analogue now covalently bound to its direct binding targets. An avidin column captured all of these biotin-decorated proteins and then in-column proteolysis released peptides, which were identified by using MS. The results were startling. From a total of about 4,500 proteins identified by MS in crude synaptosomes, about 12% (540) were captured by using the click strategy, and, of these, about 200 were deemed to be “propofol specific” on the basis of propofol protection assays. Although many captured proteins may be simply associated with propofol targets and not themselves true propofol-binding targets, it is clear that propofol is a promiscuous drug, and dozens and perhaps hundreds of molecular targets are influenced at clinical concentrations. In contrast to the results of the simple “shotgun” approach, several of the captured proteins were low-abundance ion channels and, gratifyingly, several synaptic GABA _A_R subunits. This is the first demonstration of direct propofol binding to GABA _A_R when still in its native synaptic environment. Curiously, only α and β subunits were captured, despite the relative abundance of the γ subunit in synapses. Subsequent molecular dynamic simulations provided a plausible explanation. The γ–β and γ–α interfaces were overly hydrated as compared with the α–β ones, indicating that water was competing with binding of the propofol analogue. This aligns with results discussed above in that the resulting asymmetric binding pattern around the heteropentamer ( Figure 1) should be more functionally provocative than a symmetric one where all interfaces are occupied ^51^. Moreover, this implies the importance of H-bonding within the interfacial sites, a hypothesis directly tested with a propofol variant that replaced the hydroxyl with an isosteric fluorine. The resulting “fropofol” molecule has physicochemical properties very similar to those of propofol but no anesthetic or sedative properties whatsoever. This highlights the importance of the hydroxyl in functionally relevant targets ^60^ like the GABA _A_R. Fropofol should be distinguished from other non-immobilizers (for example, the cyclobutanes ^61^), as those molecules had such pronounced physicochemical differences that a lack of activity was almost certainly due to very low water solubility. Fropofol, however, still has significant effects on myocardial (but not skeletal) contractility, indicating that these targets lack an important H-bond in their sites ^60^. This is one of the first demonstrations that primary and side effects are separable through fairly minor changes to the alkylphenol molecule.

### Voltage-gated ion channels

Monovalent voltage-gated ion channels (VGICs), which are necessary for setting membrane potential and the initiation and propagation of action potentials, are plausible targets of anesthetics. In general, potassium channels are relatively insensitive to propofol ^62^, although most inhalational anesthetics and alcohols inhibit them ^63^. Sodium channels, on the other hand, appear to be inhibited by clinically relevant propofol concentrations ^64^. The precise mechanisms are not yet clear, but evidence for anesthetic sites on these VGICs exists. Propofol and sevoflurane sites in the simpler Kvx channels have been revealed by photolabeling studies ^62, 65^, while evidence for propofol sites in the more complex Navx channels has been largely through molecular modeling and mutagenesis, although isoflurane sites have been revealed via nuclear magnetic resonance studies ^66^. The data suggest at least two general classes of anesthetic sites. First, binding to an important “hinge” site, located between the voltage sensor and the pore domain (S4–5 linker) at the intracellular membrane surface, can alter activity in either direction ^63^, and, second, the pore domain itself.

The pore is an amphiphilic environment fairly well suited to anesthetics, occupancy of which would be expected to produce inhibition. The direction of modulation in these many examples of Kvx and Navx will then depend on relative affinity, and therefore occupancy, of each of these sites.

### HCN-1

The importance of the hyperpolarization-activated cyclic nucleotide-regulated (HCN) channels to propofol actions was demonstrated by a diminished sensitivity to propofol in HCN1-knockout mice ^67^. Moreover, HCN channels are thought to, in part, underlie the afferent hyperexcitability resulting in neuropathic pain. Propofol and related alkylphenols inhibit the HCN-1 channel at subhypnotic concentrations, making the alkylphenol a reasonable chemotype for further medicinal chemistry to create an analgesic drug with less hypnotic potency ^68– 71^. This recent work suggests that increasing the bulk and hydrophobicity of the alkylphenol side chains (for example, 2,6-Di-tert-butylphenol) accomplishes this to some degree, lending confidence that an entirely novel class of analgesic, useful for neuropathic pain, may emerge from the alkylphenol chemotype ^72^. Binding sites and mechanisms by which the alkylphenols inhibit this channel are not yet clear, but the recent publication of a cryo-electron microscopy structure ^73^ should facilitate work in the area.

### Kinesin

Building on work over the previous two decades which implicated actin, tubulin, and other cellular or synaptic cytoskeleton and transport machinery in general anesthetic action ^74– 76^, investigators recently examined the effects of propofol on the anterograde motor kinesin. Kinesin is like a locomotive that runs along microtubule “tracks” by using its dimeric motor heads to step from β to β tubulin subunit ^77^. Should these tracks be decorated with bound anesthetic molecules, it is plausible that motor function and ultimately transport would be altered. In single-molecule experiments, where, instead of its usual cargo, kinesin is attached to a fluorescent bead and microtubules are immobilized on a substrate, it was noticed that clinically relevant concentrations of propofol reduced run length by half and had no effect on velocity ^78^. In other words, the train goes just as fast but derails. This might indicate extensive tubulin binding of propofol, but could also be due to effects within kinesin itself. If the latter, the single-molecule results suggest that the propofol site is allosteric to the nucleotide-binding site and that it is not influencing ATPase activity. Most likely, an allosteric site in kinesin is formed when the motor head binds to β tubulin. In either case, though, the result is the same, and cargo is transported by these anterograde kinesins reaching their destinations more slowly or not at all. Depending on redundancy, this may have important consequences in terms of the critical timing of cellular/neuronal activity. Applying the anesthetic-null variant fropofol (see above) had no effect, lending some credence to the possibility that kinesin modulation underlies propofol-induced unconsciousness. It is also plausible that an interaction between kinesin and propofol underlies a number of propofol’s less desirable actions, such as neurotoxicity at the extremes of age ^79^.

### TRPx receptors

**Figure 2.**
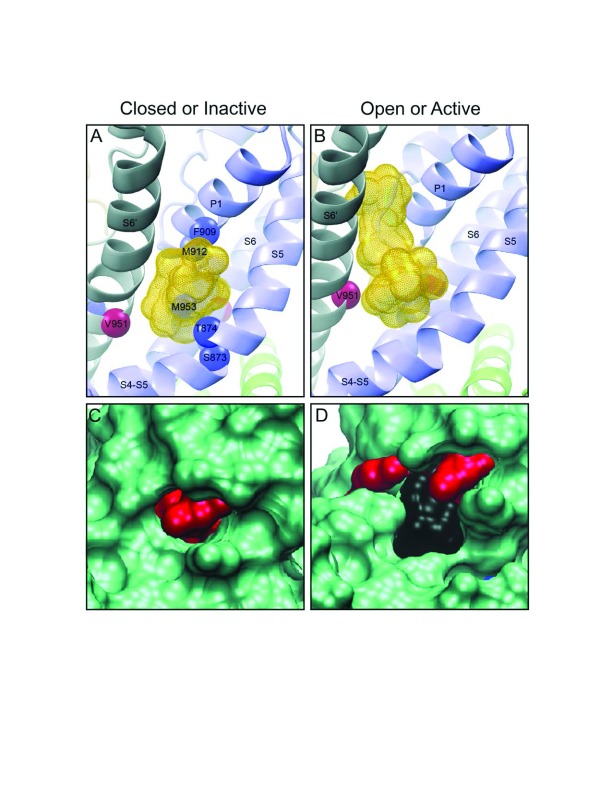
Recent examples of propofol-binding proteins. In both the TRPA1 ( **A**, **B**) and SIRT2 ( **C**, **D**), note that the propofol-enhanced active or open state ( **B**, **D**) suggests that the alkylphenol-binding pocket is actually enlarged somewhat as compared with the closed or inactive state ( **A**, **C**). We believe that this is an example of enhanced affinity as a result of reduction in the entropic penalties of binding and may be a common feature in conformationally sensitive binding, a form of “induced fit”. The yellow stippled shapes in ( **A**) and ( **B**) represent multiple poses of both propofol and azipropofol when docked in the vicinity of the photolabeled residues. In ( **C**) and ( **D**), the red shapes are photolabeled residues and the black shape is the surface rendering of the cavity occupied by propofol.

It is well known that propofol both causes pain on injection and sensitizes nociception. The candidate molecular targets for transducing these effects are the widely distributed TRPA and TRPV receptors ^80, 81^. TRPA-1, in particular, is known to be modulated by propofol in a biphasic manner: activated at low, clinically relevant concentrations and inhibited at higher concentrations. The mechanism or mechanisms by which propofol produces these actions are suggested by recent mutagenesis ^82^ and photolabeling results with aziP *m* ^83^. For example, the canonical “hinge” region of these channels, connecting sensor to pore domains and typically located in and around the cytoplasmic face of the transmembrane region, forms a binding site whereby, when occupied, the open state is stabilized, enhancing current flow ( Figure 2). However, when the channel is in the open state, a lower-affinity pore site is created which becomes progressively occupied as the propofol concentration is raised, reducing current flow. This sequential occupancy of two sites of differing affinity and effect is presumably responsible for the bimodal modulation of the TRPA-1 receptor and may be a general model for actions on other channels. Since activation of a nociceptor is not a desirable attribute of a general anesthetic, it would be important to determine characteristics of the binding site in order to use medicinal chemistry approaches to weaken binding to this activating site. This may not only reduce pain of injection but also increase anesthetic potency, depending on the central nervous system (CNS) distribution of the TRPA-1 receptor.

### SIRT-2 deacetylase

On evaluation of the propofol-binding proteome of various CNS tissues, it was found that myelin contained a highly specific protein target of propofol that was ultimately determined to be the SIRT-2 deacetylase ^84^. The acetylase and deacetylase enzymes are key intracellular regulators of a wide variety of events ^85^; thus, an effect of anesthetics would be expected to have far-reaching consequences. Since a binding target does not necessarily indicate a functional target, a number of experiments were conducted to determine whether propofol had an effect on SIRT-2 deacetylase activity and at a clinically relevant concentration. Indeed, when isolated SIRT-2 was exposed to low-micromolar concentrations of propofol, its activity was significantly inhibited, and the structural mechanism was characterized ( Figure 2) ^84^. Though unlikely to underlie the primary, desired action of propofol, this interaction may underlie one or more of the many side effects of propofol. Since acetylation and methylation systems are responsible for epigenetic regulation, this interaction could even be responsible for delayed or persistent effects of propofol, such as developmental neuromodulation ^79^.

## Summary

Much progress has been made in understanding the mechanisms of propofol’s actions on its canonical molecular target, the GABA _A_R, but a role for other molecular targets is gaining momentum. Propofol is undeniably a promiscuous anesthetic ligand, and its diverse molecular targets are integrated in a manner that is still poorly characterized. Finally, evidence suggests that further medicinal chemistry of the alkylphenol chemotype may allow enhancement of selected actions.
